# An ultrafast-response and flexible humidity sensor for human respiration monitoring and noncontact safety warning

**DOI:** 10.1038/s41378-021-00324-4

**Published:** 2021-11-29

**Authors:** Xiaoyi Wang, Yang Deng, Xingru Chen, Peng Jiang, Yik Kin Cheung, Hongyu Yu

**Affiliations:** Department of Mechanical and Aerospace Engineering, Hong Kong University of Science and Technology, 999077 Kowloon, Hong Kong SAR China

**Keywords:** Sensors, NEMS

## Abstract

The humidity sensor is an essential sensing node in medical diagnosis and industrial processing control. To date, most of the reported relative humidity sensors have a long response time of several seconds or even hundreds of seconds, which would limit their real application for certain critical areas with fast-varying signals. In this paper, we propose a flexible and low-cost humidity sensor using vertically aligned carbon nanotubes (VACNTs) as electrodes, a PDMS-Parylene C double layer as the flexible substrate, and graphene oxide as the sensing material. The humidity sensor has an ultrafast response of ~20 ms, which is more than two orders faster than most of the previously reported flexible humidity sensors. Moreover, the sensor has a high sensitivity (16.7 pF/% RH), low hysteresis (<0.44%), high repeatability (2.7%), good long-term stability, and outstanding flexibility. Benefiting from these advantages, especially the fast response, the device has been demonstrated in precise human respiration monitoring (fast breathing, normal breathing, deep breathing, asthma, choking, and apnea), noncontact electrical safety warning for bare hand and wet gloves, and noncontact pipe leakage detection. In addition, the facile fabrication of the flexible platform with the PDMS-Parylene C double layer can be easily integrated with multisensing functions such as pH sensing, ammonium ion sensing, and temperature sensing, all of which are useful for more pattern recognition of human activity.

## Introduction

Humidity sensing is essential in broad applications, including process control in industry, medical facilities, environmental monitoring of greenhouses, cleanrooms, and heating, ventilation, air conditioning (HVAC) systems^1–8^. Based on different sensing mechanisms and readout signals, humidity sensors can be categorized into several types, including resistance^2,9^, capacitance^10^, optical type^11^, surface acoustic wave^5^, impedance^12^, and field-effect transistor^13^. In addition, the sensing materials are also diverse, such as metal oxides^14,15^, carbon nanotubes^16^, graphene^17^, graphene oxide^12,18^, hydrogels^19^, and polyimides^20^. With the development of flexible and soft electronics, in recent years, flexible humidity sensors have become an essential component for wearable applications^21–32^. For example, Zhu^33^, Duan^28^, and Alrammouz^34^ reported a cost-saving, environmentally friendly paper-based flexible humidity sensor and demonstrated its applications for breathing, noncontact sensing, skin humidity detection, etc. Komazaki^35^, Ma^36^, and Rauf^37^ incorporated the sensing materials of PDMS-CaCl_2_, polyimide, and metal-organic frameworks with flexible textile substrates for humidity sensing. Zheng^25^ fabricated a transparent and flexible humidity sensor using biocompatible, natural biomaterial silk fibroin films as the substrate. In addition to the above paper and silk fibroin substrates, other flexible materials are also being explored as substrates for flexible humidity sensors, including polyimide^27^, PDMS^38^, cellulose/KOH composites^39^, polyethylene terephthalate (PET)^40^, and polycarbonate (PC)^15^. In addition, to be incorporated with flexible substrates, metals^41^, graphene^42^, ITO^43^, and graphite^44^ have been investigated as sensing electrodes for humidity sensors. Most of the electrodes are directly deposited or printed on the surface of the flexible substrate, and the adhesion performance is not robust and easily peels off at the crease area. Furthermore, since electrode materials are normally inorganic rigid materials, they tend to be damaged during bending or other deformations. The common solution is to put the electrode layer in the neutral plane to reduce the stress issue^45,46^. However, it cannot always be realized given the device structure, especially when two layers of electrodes are needed. Moreover, when targeting wearable electronics applications, flexibility should only be the prerequisite, other merits (e.g., high sensitivity, large measurement range, fast response, high stability, low-temperature independence) are still vital. However, most of the current works focus on enhancing the sensitivity and changing different materials for better flexibility^29,33,34^. Very few works have put effort into improving the response time. Kano^24^ and He^47^ proposed two types of flexible humidity sensors with fast response/recovery times of approximately several tens of milliseconds using sensing materials of silicon-nanocrystal films and tunable graphene polymer heterogeneous nanosensing junctions, respectively. For one of the most important applications of humidity sensors, respiration monitoring, the breathing rate of coughing, asthma, and choking is much higher than the normal respiration frequency (~0.33 Hz). The humidity sensor with a slow response would not catch the sudden change of breathing signal^48^. In addition, although some RH sensors with a slow response could still sense fast-breathing behavior with their transient response, the information they offer is usually only the respiration strength. For example, since the humidity level is nearly the same for different breathing conditions (normal, fast, and deep breathing conditions), sensors with larger response times can sense changes in respiration, but the sensor signal variation amplitudes between fast breathing, normal breathing, and deep breathing would be quite different^29,49^. In addition, due to the accumulation effect, for the fast-breathing signal, the overall change in the record data has a distinct drift^29^. Moreover, for some fast-breathing conditions, the monitoring signal will have some sharp corners because the sensor cannot be efficient enough to precisely follow the breathing signals and catch clear breathing patterns^36^. They cannot truly provide the whole waveform of the breath, so they cannot precisely indicate the real respiration condition^29^.

Herein, for the first time, we present a flexible relative humidity sensor integrating a PDMS-Parylene C double-layer thin film as the flexible substrate, vertically aligned carbon nanotubes as the electrodes, and graphene oxide as the sensing material. VACNTs are stuck into flexible substrates and have ultra-robust performance under deformation and folding. The sensor achieves an ultrafast response, high sensitivity, good repeatability, and good long-term stability as a whole application package. In addition, a high-performance relative humidity sensor is demonstrated in real applications for human respiration monitoring, pipe leakage detection, and noncontact electric safety warning, showing its potential as a flexible or curved humidity-sensing node in wearable electronics or industry monitoring.

## Materials and methods

### Preparation of the VACNT Electrode

**Fig. 1:**
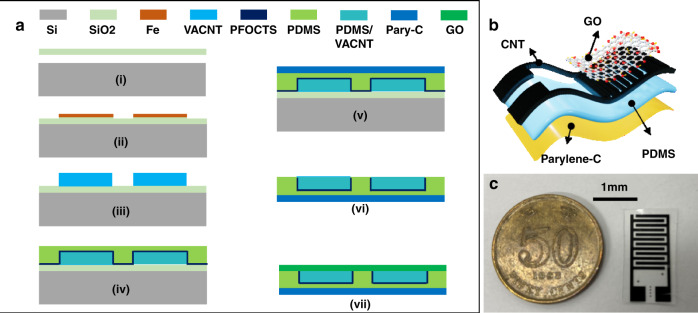
Schematic drawing of the flexible humidity sensor. **a** Fabrication process of the flexible humidity sensor. **b** Exploded view of the flexible sensor. **c** The image of the fabricated RH sensor (Pary-C means Parylene C)

The detailed fabrication process is shown in Fig. 1a. A 2-nm Fe thin seed layer was grown on a silicon wafer with a 1-µm silicon oxide isolation layer on top (Fig. 1a(i) using an E-beam evaporator and patterned for an interdigital finger shape with a lift-off process (Fig. 1a(ii)). Then, the VACNT forest (Fig. 1a(iii)) was synthesized using the microwave plasma-enhanced chemical vapor deposition (PECVD) apparatus to form the sensor electrodes.

### Preparation of the flexible substrate

Polydimethylsiloxane (PDMS) and Parylene C were used to fabricate the double-layer flexible substrate, which was used to peel off the VACNT electrodes from the silicon wafer. The PDMS and Parylene C dimers were purchased from Dowsil and Galentis, respectively. To reduce the PDMS adhesion to the silicon substrate and ensure the integrity of the PDMS-Parylene C film during the peeling-off process from the Si wafer, 100 μl trichloro (1H,1H,2H,2H-perfluorooctyl) silane (PFOCTS) (Merck & Co.) was evaporated and coated onto the surface of the wafer using the following procedure. The wafers and opened PFOCTS container were put into the same vacuum chamber, which was pumped for 5 min to reach base vacuum pressure and kept sealed. It took 3 h for PEOCTS to evaporate from the container and coat onto the wafer surface. After the surface treatment, a degassed PDMS precursor mixer (monomer/curing agent: 10/1) was spun on the surface of the wafer to cover the VACNTs using a spin coater with a rotation speed of 500 rotations/min for 40 s (Fig. 1a(iv)). The thickness of the PDMS was 140 µm. Then, the 20-µm Parylene C layer was coated using chemical vapor deposition (Fig. 1a(v)). After that, the thin film was peeled off (Fig. 1a(vi)) and cut into individual sensor pieces.

### Preparation of the GO solution and sensing film

Graphene oxide (GO) as a sensing material was used in the devices (Fig. 1a(vii)), which was purchased from Sigma Aldrich company with an initial concentration of 4 mg/ml. Three kinds of diluted solutions (using deionized water to dilute) were synthesized with GO concentrations of 1, 0.5, and 0.25 mg/ml. A 30 μl GO solution was dripped and coated on the VACNT electrodes of the sensor using pipettes. To accelerate the drying process, the sensor was baked on a hot plate at a temperature of 70 °C for 10 min and was completely fabricated. Figure 1b shows an exploded view of the flexible humidity sensor with four layers, including Parylene C, PDMS, VACNT, and graphene oxide. The image of the real RH sensor is illustrated in Fig. 1c.

### Characterization of VACNT and GO material

**Fig. 2:**
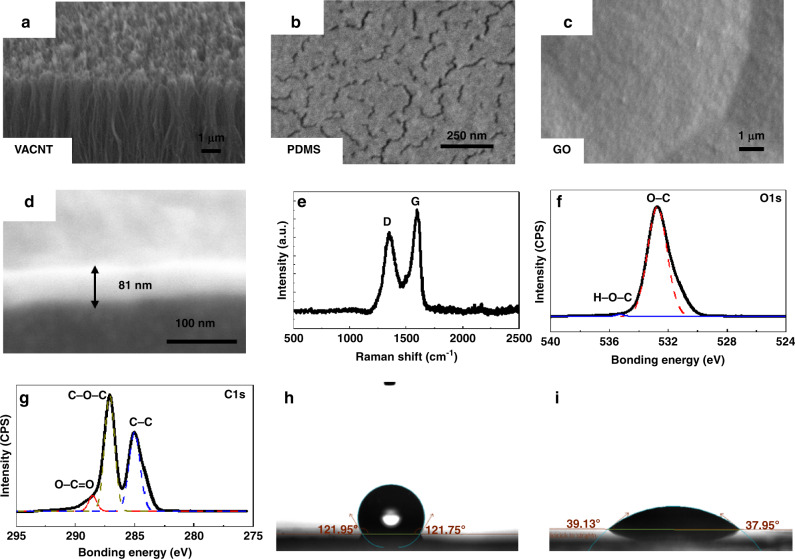
Characterization of electrode material, sensing material, and substrate. **a** SEM image of the cross-sectional view of vertically aligned carbon nanotubes. **b** SEM image of the top view of the VACNT-PDMS mixture surface. **c** SEM image of the top view of GO surface. **d** SEM image of the cross-sectional view of the GO film. **e** Raman spectrum of GO film. **f**, **g** XPS fully scanned spectra of the graphene oxide of O 1 s and C 1 s. **h**–**i** The surface contact angles of the flexible substrate without GO and with GO

The VACNTs (Fig. 2a), pure flexible substrate (Fig. 2b), and graphene oxide sensing film (Fig. 2c) were characterized using scanning electron microscopy (SEM). VACNTs suck into flexible substrates, which can be much more robust. The stability of the electrodes under deformation and high pressure is characterized (Supplementary Fig. S1), which shows low resistance variation (<1%). The thickness of the graphene oxide coating (Fig. 2d) was 81 nm with a diluted GO solution of 0.25 mg/ml (30 μl). The thicknesses of GO solutions of 0.5 mg/ml and 1 mg/ml were 276 nm (Supplementary Fig. S2a) and 534 nm (Supplementary Fig. S2b), respectively. Raman spectrum analysis was conducted for graphene oxide characterization with two obvious peaks corresponding to the D-band (1351 cm^−1^) and G-band (1596 cm^−1^) (Fig. 2e), indicating the rich chemical functionalization of the graphene oxide structure. The chemical composition of the GO surface was characterized by X-ray photoelectron spectroscopy (XPS), illustrating the expected elemental peaks of C and O. The binding energy of the O 1 s spectrum can be assigned to H–O–C and O-C species (Fig. 2f), while C 1 s is decomposed into C–C, C–O–C and O–C = O groups (Fig. 2g). The XPS analysis results show that GO contains rich hydrophilic species, contributing to its superb water adsorption ability and good humidity-sensing performance. The hydrophobic performance was tested using a contact angle meter (Biolin Theta). Figure 2h shows the hydrophobic property (121.75°@10 s) of the VACNT electrode surface on a PDMS-Parylene C double-layer flexible substrate, and Fig. 2i shows the hydrophilic performance (39.13°@10 s) of the flexible sensor with a GO layer on top of VACNTs. The hydrophilic property of the GO thin film makes it suitable for water molecule condensation.

## Results and discussion

### Performance characterization

**Fig. 3:**
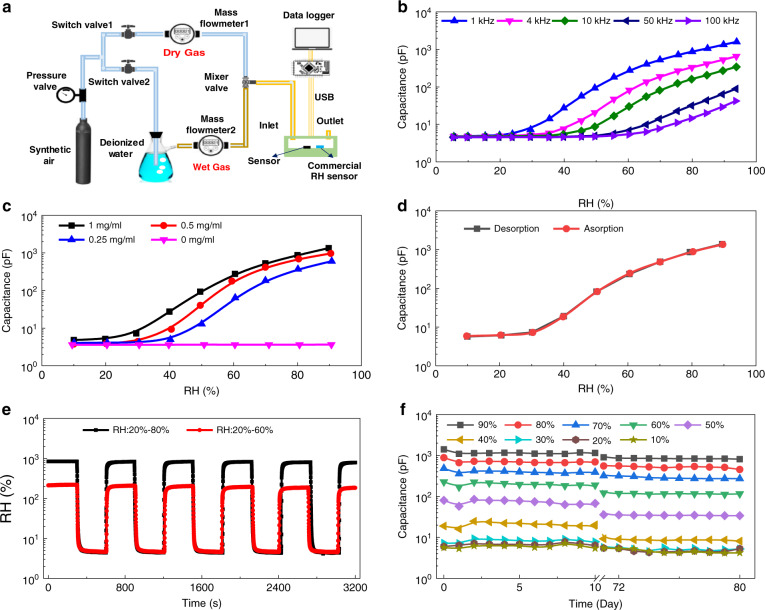
Performance characterization of the flexible humidity sensor. **a** Schematic of the homemade test setup for the base performance characterization of the flexible humidity sensor. **b** Capacitance change of the flexible humidity sensor with the RH change from 5 to 95% at different readout circuit working frequencies. **c** The effect of GO coating film thickness on the sensing performance. The GO films were created by three types of solutions (1, 0.5, 0.25, and 0 mg/ml) for different sensing layer differences. **d**–**f** Hysteresis, repeatability, and long-term stability performance characterization of the flexible humidity sensor

Before investigating the various potential applications of the flexible relative humidity sensor, the device was tested with a homemade testing setup (Fig. 3a) to characterize the sensing performance. The synthetic dry air (oxygen 21%, nitrogen 79%, H_2_O < 5 ppm) was divided into two channels and controlled with two mass flow meters. One channel worked as the source of dry air and another was wetted through the deionized water as a moisture source. A reference relative humidity sensor (Sensirion, RH_C-SHT20) was mounted into the testing chamber aside from our device. Arduino Yun board and RS LCR meter were used to record the reference RH value and capacitance of the flexible sensor, respectively. Figure 3b–f shows the primary performance of the humidity sensor with the *y* axis scaled as a logarithm. Figure 3b compares extracted device capacitance under different AC signal frequencies (1, 4, 10, 50, 100 kHz). It reveals that a lower excitation frequency will provide a higher signal output. Further, the sensing output is not linear with the relative humidity, indicating that the sensor capacitance shifts higher monotonically with the relative humidity increase. It can be explained that at low relative humidity, water molecules are primarily physiosorbed onto the available active sites of the GO surface through double hydrogen bonding^42^. Besides, the absorbed water molecules are beneficial in strengthening the polarization effect and increasing the dielectric constant, leading to an increase of sensor capacitance with rising RH^21^. The high sensitivity of 16.7 pF/% RH was achieved at the excitation frequency of 1 kHz referring to the relative humidity change from 10% (4.84 pF) to 90% (1340.2 pF). Flexible sensors with different film thicknesses were achieved by three types of GO concentrations in the diluted GO solutions (1 mg/ml, 0.5 mg/ml, and 0.25 mg/ml). Figure 3c depicts the performance of flexible sensors with and without GO at the working frequency of 1 kHz. According to the hydrophobic performance of pure flexible substrate shown in Fig. 2h, the sensor without GO is not sensitive to the humidity change and shows a stable capacitance value of 3.6 pF. In addition, the small variation (0.04 pF) of the humidity-sensing performance of the pure substrate can be neglected concerning its effects on the capacitance change of the sensor with GO. The sensitivity of these three types of sensors is 16.7 pF/% RH (1 mg/ml), 12.17 pF/% RH (0.5 mg/ml), and 7.467 pF/% RH (0.25 mg/ml) at 1 kHz, respectively. The hysteresis performance of the sensor made with the solution GO concentration of 1 mg/ml is characterized as shown in Fig. 3d. The hysteresis value *H*_*RH*_ was calculated by the following equation

1 $${{{\mathrm{H}}}}_{RH} = \frac{{C_{D\_RH} - C_{A\_RH}}}{{S_c}}{{{\mathrm{\% }}}}$$

where *C*_*D_RH*_ and *C*_*A_RH*_ are the capacitance values tested at certain RH values in the desorption and absorption process, respectively, and *S*_*c*_ is the sensitivity of the device. A low hysteresis of the flexible humidity sensor was achieved, which was smaller than 0.44%. The larger hysteresis mainly occurred in the high humidity regime, which may be caused by the residual water molecules in the bottom part of the graphene oxide^5^. The repeatability (Fig. 3e) was tested with the periodical change (with a period of 5 mins) of the supply wet gas for two-step signals (20% RH-80% RH and 20% RH-60% RH). The repeatability index *C*_*r*_ can be expressed as

2 $${{{\mathrm{C}}}}_r = \frac{{\left| {C_{peak} - C_{peak\_average}} \right|}}{{C_{range}}}{{{\mathrm{\% }}}}$$

where *C*_*peak*_ and *C*_*peak_average*_ are the peak capacitance and its average value at each period, respectively, and *C*_*range*_ is the capacitance difference at two RH stages. Based on this definition, the variation in the repeatability of the device is calculated with maximum *C*_*r_max*_ values of 2.7% and 2.1% for 20% RH-80% RH and 20% RH-60% RH step signals, respectively. To check the stability of the flexible humidity sensor, the sensors were first tested for ten days and then left for two months, followed by an additional 10 days of testing. The results are shown in Fig. 3f. The sensor can maintain better short-term stability for all levels (10– 90%) of humidity, while sensitivity attenuation exists from a long-term view, which will be the focus of future work.

**Fig. 4:**
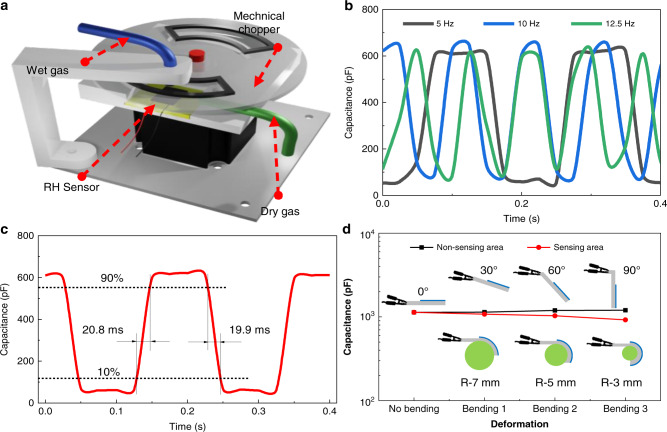
Response time and deformation characterization. **a** Schematic of the test setup for response time characterization. **b** Testing results of the capacitance change of the flexible humidity sensor under different switching frequencies of the mechanical chopper. **c** Response and recovery characterization. **d** Deformation test of the flexible sensor under bending nonsensing areas and sensing areas

For the response time characterization, the test setup shown in Fig. 3a is not adequate for accurate measurement because the wet gas flowing through the pipe and the stabilization process of the relative humidity in the testing chamber take a considerably longer time than the response time of our devices. To solve this problem, a homemade characterization setup (Fig. 4a) was created specifically for the response time measurement. A mechanical chopper driven by a step motor was used to switch the wet gas and change the local relative humidity. Three types of switching frequencies, including 5, 10, and 12.5 Hz were tested. Figure 4b shows that the sensor could not reach the saturation plateau when the switching frequencies were 10 and 12.5 Hz, while the sensor shows saturation under a slower switching frequency of 5 Hz. Figure 4c shows that the sensor has an ultrafast response (20.8 ms) and recovery (19.9 ms) by calculating the response signal from 10% *C*_*range*_ to 90% *C*_*range*_. The fast response results from many reasons. The first reason is that the sensing material GO film contains very rich functional groups, including H–O–C, O–C, C–C, C–O–C, and O–C=O groups, which contribute to its superior water adsorption ability. Second, the hydrophilic property of the GO thin film makes it much more suitable for water molecule condensation. Third, the thin GO film is also a critical factor to improve the sensor response performance; the thinner the film is, the faster the sensor response^50^. The response/recovery time for the thicker GO films was also found to be 190 ms/440 ms (0.5 mg/ml) (Supplementary Fig. S3a) and 431 ms/647 ms (1 mg/ml) (Supplementary Fig. S3b). To check the response and hysteresis stability, the device was examined again on the 75th day, the response time (Supplementary Fig. S4a) was still at the same level of ~20 ms, and the hysteresis (Supplementary Fig. S4b) also did not change much, with a value of 0.81%.

As a flexible sensor, the performance under static deformation was tested by bending both nonsensing and sensing areas under a circumstance with an RH of 85%. Figure 4d shows that the capacitance variation is negligible under small deformation when bending the nonsensing area. The capacitance decreases when there is larger bending at the sensing area, which is caused by microcracks during this process^51,52^. The smaller the bending radius is, the larger the capacitance drop, such as a 5% drop for a 7-mm bending radius, 9.1% for a 5-mm bending radius, and 18.7% for a 3-mm bending radius. The capacitance changes of the humidity sensor under the deformation and recovery process (radius of 3 mm) are shown in Supplementary Fig. S5a. The hysteresis is also characterized under deformation conditions. Supplementary Fig. S5b shows that the hysteresis (1.8%) under deformation conditions is slightly larger than that of the initial state (0.44%). However, the sensor recovers its initial performance after returning to the flat state, and the sensor’s performance can maintain stability under bending conditions, which will be demonstrated in the respiration monitoring experiment section (bending radius is 9 mm).

The temperature effect is also characterized, as shown in Supplementary Fig. S6. As the temperature increases, air can hold more water molecules, so the capacitance of the humidity sensor will also increase. The sensor was characterized at two humidity levels (60% RH and 80% RH) with a temperature range from 25 to 55 °C. The average temperature coefficient of the capacitance of the sensor is 0.01/°C. GO is rich in oxygen-containing functional groups, which are sensitive to volatile organic compounds (VOCs). We built a homemade chamber (Supplementary Fig. S7) to test the sensor response of acetone (Supplementary Fig. S8a) and isopropyl alcohol (IPA) (Supplementary Fig. S8b). As the sensor is proposed for respiration monitoring, the carbon dioxide concentration in exhaled breath is larger than that of inhaled breath, and the carbon dioxide effect on the sensing performance of the sensor is also tested. Supplementary Fig. S9 shows the pure carbon dioxide effect on the sensitivity of the sensor, which is ~1.5 times larger than that of synthetic air at a humidity of 90% RH. Based on the measurement results, we calculate the sensing performance of the humidity sensor for exhaling breath with 4% CO_2_, and the results show that the largest capacitance change of the sensor for exhaling breath with up to 4% CO_2_ is only 1.8%. Finally, a comparison table of different humidity sensors is summarized (shown in Table 1).

**Table 1:** Summary of the characteristics of different humidity sensors

Ref.	Sensing type	Sensing material	Electrodes	Substrate	Response/r.ecover	Sensitivity
This work	Capacitive	GO	VACNT	Pary-C /PDMS	20.8 ms/19.9 ms	16 pF/%RH
^25^	Resistive	Silk fibroin	silver	PET	73.1 s/11.3 s	~0.2 nA/%RH
^21^	Capacitive	rGO/SnO_2_	Cu/Ni	PI	65 s/67 s	1605pF%RH
^42^	Capacitive	GO	LIG	PI-PET	15.8 s/NA	3215 pF/%RH
^24^	Resistive	Si NCs	Gold	PI	~40 ms	N/A
^53^	Capacitive	Paper	Aluminum	MP	266 s/<10 s	83.2fF/%RH
^47^	Resistive	GNCP	Gold	PI	20 ms/17 ms	20000*

*It is defined by the equation (*R*_*RH*_ − *R*_*0*_)/*R*_*0*_ in the reference paper.

### Respiration test

**Fig. 5:**
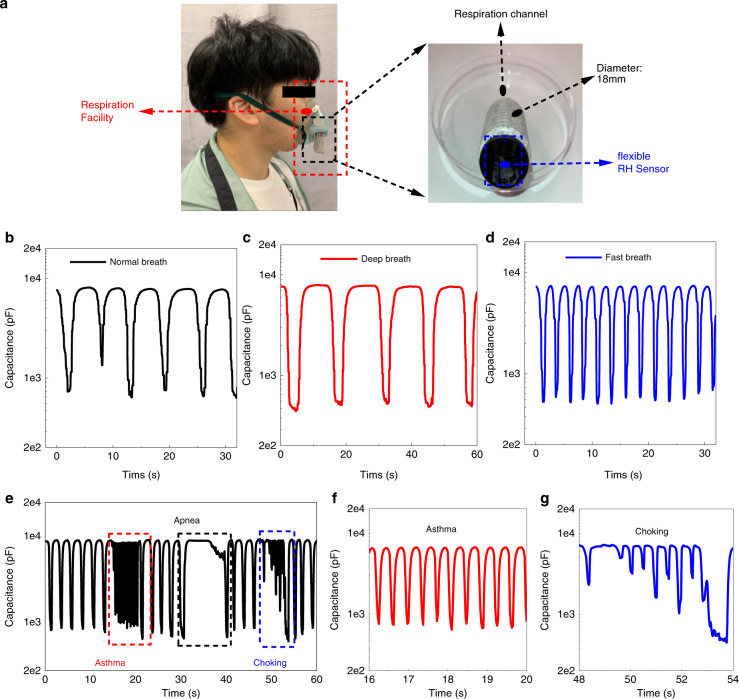
Human respiration testing. **a** Images of the test installation of the respiration facility and the location of the flexible humidity sensor. **b**–**d** The respiration testing results of normal breath, deep breath, and fast breath. **e** The main characteristics of abnormal breaths, including asthma, apnea, and choking, are mimicked and recognized by our ultrafast flexible humidity sensor with better feature patterns. **f**, **g** Enlarged graph of the asthma breath pattern and the choking breath pattern

Respiration is an important vital signal for health status monitoring. Most reported sensors could not follow the sudden change (>1 Hz) of the breathing signal due to their slow response time. Based on the ultrafast response of the proposed flexible humidity sensor, it is practical to employ this sensor for monitoring respiration precisely, especially since it can capture comprehensive information for abnormal conditions, such as choking, apnea, and asthma. The flexible sensor was mounted inside of the hollow cylinder (inner diameter of 18 mm, i.e., the bending radius of 9 mm) with a double side adhesive (Fig. 5a). The respiration facility was the normal type used in medical treatment and can conformally attach to the surface of the human face, as illustrated in Fig. 5a. The normal breath (0.16 Hz, Fig. 5b), deep breath (0.08 Hz, Fig. 5c), and fast breath (0.38 Hz, Fig. 5d) were tested with our relative humidity sensor, which showed that all the breathing patterns were clearly captured without losing features. In addition, the main characteristics of abnormal breathing conditions, including choking, apnea, and asthma (~3 Hz), were mimicked and recognized by our ultrafast flexible humidity sensor with precise feature patterns^54^ (Fig. 5e–g).

### Pipe leakage test

**Fig. 6:**
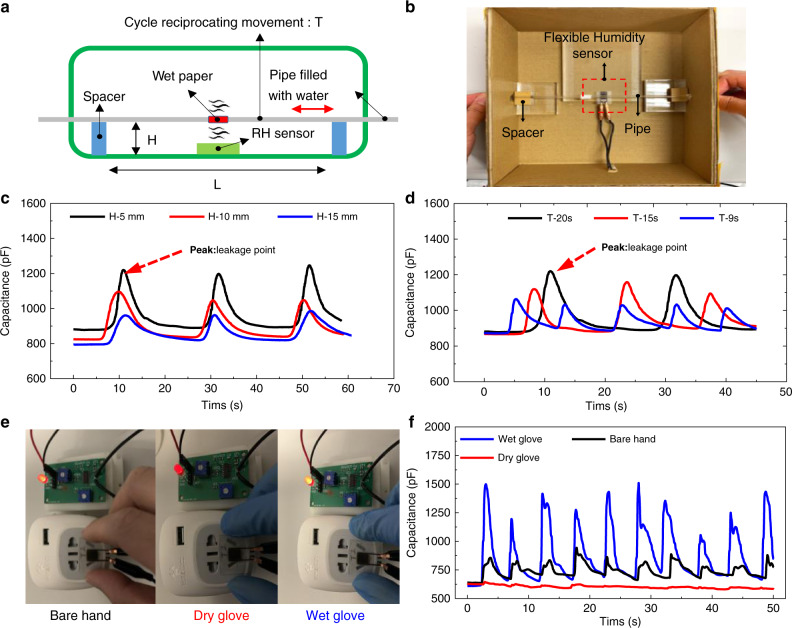
Noncantact humidity sensing testing. **a** Schematic of the pipe leakage testing method. **b** The image of the test setup. The results of leakage detection using the flexible sensor with (**c**) different spacer heights and (**d**) different moving speeds of the pipe. **e** Images of the testing for electric safety warning using the flexible humidity sensor under the condition of a bare hand, a hand with a dry glove, and a hand with a wet glove. **f** Test results of the flexible humidity sensor’s response to three touching conditions of the electric adapter

Pipe leakage tests are important for fluid pipelines, especially for water pipelines in the tunnels of subways and other difficult inspection areas. Water leakage can change the humidity, which can be monitored by the humidity sensor, and a warning signal can be sent to the control center to ensure safety and prevent property damage. We provide a simple testing method to demonstrate this pipe leakage detection application shown in Fig. 6a employing our humidity sensor. A rectangular box with a cover was used for the testing, and a 4-mm PET pipe filled with water mimicked the water supply pipe (Fig. 6b). A small hole was created at the central part of the pipe, and clean paper was entangled at the location of the hole as a water reservoir that can provide continuous water molecules into the environment. The humidity sensor was attached to the bottom of the box, and the pipe was supported by two spacers at both sides of the box with a height of H. The pipe was moved periodically with a constant speed and a period of T. The flexible sensor sensed the humidity change when the leakage point of the pipe moved closer to or away from the humidity sensor. Figure 6c shows that a lower spacer height enhanced the sensing performance since the water molecular density in the air is dependent on the distance based on the reported touching testing results^10,42^. The peak capacitances for different spacer heights are 1.22 nF (5 mm), 1.01 nF (10 mm), and 0.96 nF (15 mm). Moreover, the pipe movement time was also tested with different moving periods, including 9 s, 15 s, and 20 s, at the same space height of 5 mm. As the moving speed increased, the peak values decreased from 1.22 nF (20 s) to 1.12 nF (15 s) and then to 1.06 nF (9 s), as shown in Fig. 6d. Compared with the ultrafast response, the decreasing response value mainly results from the limited water molecular diffusion speed to its surrounding circumstance.

### Electric safety test

Electric safety is not only an important aspect in the industry but also a critical issue in family and public areas. Current electric safety detection technology is based on signals such as leakage current and current overload generated after the event to identify the accident. We propose a novel method to monitor dangerous situations where people approach electric adapters. The flexible sensor can be easily attached to the surface of the electric adapter shown in Fig. 6e. A bare hand, a hand wearing a dry glove, and a hand wearing a wet glove are tested to check the response of the flexible sensor periodically approaching the electric adapter. Figure 6f illustrates that the diffusion of the surface sweat from the bare hand influences the local humidity and causes a sudden capacitance perturbation of the flexible humidity sensor. If the hand is covered with a rubber glove, sweat diffusion will be hindered, so the sensor will not have a significant variation (~20 pF) to the approaching process of the hand. However, if the gloves are wetted with water, the sensor can identify the significant local humidity perturbation shown in Fig. 6f. Compared to the initial capacitance value (610 pF) of the flexible sensor, the peak values of the flexible sensor’s response to a bare hand and a hand with a wet glove are 857F and 1503 pF, respectively. We demonstrate that the proposed flexible humidity sensor can be used to sense the danger approaching process as an electric safety warning system integrated with a lighting diode to protect human safety.

## Conclusions

In this work, we successfully demonstrate a flexible humidity sensor using vertically aligned carbon nanotubes as robust conductive electrodes, GO as the sensing material, and a PDMS-Parylene C double layer as the flexible substrate. Its fabrication process is a novel generic method for the development of flexible sensors, which can be transferred to realizing other types of flexible sensing functions using the same platform. The device is characterized by demonstrating an ultrafast response (20.8 ms)/recovery (19.9 ms), high sensitivity (16.7 pF/% RH), low hysteresis (<0.44%), and high repeatability (2.7%). The ultrafast-response characteristic makes it feasible for the accurate sensing of human respiration, including abnormal behaviors such as choking, apnea, and asthma. Future work will focus on the deep analysis of the recorded data for medical information extraction, including breathing rate and breathing mean ventilation. Moreover, the device is also capable of leakage detection in water pipelines and electric safety monitoring, which are promising applications for personnel working in laboratories, mines, petrochemical industries, and other vulnerable industries.

## Supplementary information

Supplemental Material
