# Supplementary material for: An ultrafast-response and flexible humidity sensor for human respiration monitoring and noncontact safety warning
Source: Microsyst Nanoeng. 2021 Nov 29;7:99. doi: 10.1038/s41378-021-00324-4 (PMC8628006; doi:10.1038/s41378-021-00324-4)
Supplement: Supplementary file 1 — Supplemental Material [file 41378_2021_324_MOESM1_ESM.docx]

**Supporting Information**

# An Ultrafast-Response and Flexible Humidity Sensor for Human Respiration Monitoring and Non-Contact Safety Warning

*Xiaoyi Wang, Yang Deng, Xingru Chen, Peng Jiang, Yik Kin Cheung, Hongyu Yu**

Department of Mechanical and Aerospace Engineering, Hong Kong University of Science and Technology, Kowloon, Hong Kong SAR 999077, China

*Corresponding author: Email: [hongyuyu@ust.hk](mailto:hongyuyu@ust.hk)

Fig. S1 Electrode resistance variation of the vertically aligned carbon nanotubes under deforming and pressing condition.

Fig. S2 SEM images of GO thickness of different GO solutions: **a** 0.5mg/ml and **b** 1mg/ml.

Fig. S3 Response time characterization of humidity sensors with thicker GO sensing films for solutions: **a** 0.5mg/ml and **b** 1mg/ml.

Fig. S4 **a** Response time and **b** hysteresis characterization of the sensor over the time (@xxth day).

Fig. S5 **a** capacitance of the humidity sensor under deforming process, **b** the humidity hysteresis performance of the sensor under deformation condition (radius of 3mm).

Fig. S6 Temperature effect on the performance of the flexible humidity sensor.

Fig. S7 Schematic illustration of the VOC sensing system.

The homemade chamber is built to test the sensor response of the acetone and Isopropyl alcohol (IPA). The desired VOC concentration was obtained by injecting the required quantity of anhydrous liquid analytes into a sealed container using a syringe. The concentrations of targeted VOCs in the chamber were calculated using the following equation ^1-3^:

$$C\text{=}\frac{22.4\rho TV_{s}}{273MV}\times1000$$

where, *C* is the concentration of the gaseous VOC at the room temperature (ppm), *ρ* is the density of anhydrous liquid VOC (g·mL^-1^), *T* is the testing temperature (*K*), *Vs* is the volume of anhydrous liquid VOC (μL), M is the molecular weight of a VOC (g·mol^-1^), and *V* is the volume of the glass container (*L*) filled with the VOC.

Fig. S8 Dynamic response test of the flexible humidity sensor for VOC sensing, the continuous response of the capacitance of sensor to different **a** acetone and **b** IPA concentrations.

Fig. S9 Carbon dioxide effect on the sensing performance of the flexible humidity sensor.

References

1. Li, X., Chang, Y., Long, Y. J. M. S. & C, E. Influence of Sn doping on ZnO sensing properties for ethanol and acetone. *Materials Science Engineering: C* **32**, 817-821, doi:10.1016/j.msec.2012.01.032 (2012).

2. Ding, B., Wang, X., Yu, J. & Wang, M. J. J. o. M. C. Polyamide 6 composite nano-fiber/net functionalized by polyethyleneimine on quartz crystal microbalance for highly sensitive formaldehyde sensors. *Journal of Materials Chemistry* **21**, 12784-12792, doi:10.1039/C1JM11847A (2011).

3. Li, D. *et al.* Virtual sensor array based on MXene for selective detections of VOCs. *Sensors and Actuators B: Chemical* **331**, 129414, doi:10.1016/j.snb.2020.129414 (2021).
